# Detection and identification of human *Plasmodium *species with real-time quantitative nucleic acid sequence-based amplification

**DOI:** 10.1186/1475-2875-5-80

**Published:** 2006-10-03

**Authors:** Petra F Mens, Gerard J Schoone, Piet A Kager, Henk DFH Schallig

**Affiliations:** 1 Koninklijk Instituut voor de Tropen (KIT)/Royal Tropical Institute, KIT Biomedical Research, Meibergdreef 39, 1105 AZ Amsterdam, The Netherlands; 2 Academic Medical Centre, Division of Infectious Diseases, Tropical Medicine and AIDS, Meibergdreef 9, 1105 AZ Amsterdam, The Netherlands

## Abstract

**Background:**

Decisions concerning malaria treatment depend on species identification causing disease. Microscopy is most frequently used, but at low parasitaemia (<20 parasites/μl) the technique becomes less sensitive and time consuming. Rapid diagnostic tests based on *Plasmodium *antigen detection do often not allow for species discrimination as microscopy does, but also become insensitive at <100 parasites/μl.

**Methods:**

This paper reports the development of a sensitive and specific real-time Quantitative Nucleic Acid Sequence Based Amplification (real-time QT-NASBA) assays, based on the small-subunit 18S rRNA gene, to identify the four human *Plasmodium *species.

**Results:**

The lower detection limit of the assay is 100 – 1000 molecules *in vitro *RNA for all species, which corresponds to 0.01 – 0.1 parasite per diagnostic sample (i.e. 50 μl of processed blood). The real-time QT-NASBA was further evaluated using 79 clinical samples from malaria patients: i.e. 11 *Plasmodium. falciparum*, 37 *Plasmodium vivax*, seven *Plasmodium malariae*, four *Plasmodium ovale *and 20 mixed infections. The initial diagnosis of 69 out of the 79 samples was confirmed with the developed real-time QT-NASBA. Re-analysis of seven available original slides resolved five mismatches. Three of those were initially identified as *P. malariae *mono-infection, but after re-reading the slides *P. falciparum *was found, confirming the real-time QT-NASBA result. The other two slides were of poor quality not allowing true species identification. The remaining five discordant results could not be explained by microscopy, but may be due to extreme low numbers of parasites present in the samples. In addition, 12 *Plasmodium berghei *isolates from mice and 20 blood samples from healthy donors did not show any reaction in the assay.

**Conclusion:**

Real-time QT-NASBA is a very sensitive and specific technique with a detection limit of 0.1 *Plasmodium *parasite per diagnostic sample (50 μl of blood) and can be used for the detection, identification and quantitative measurement of low parasitaemia of *Plasmodium *species, thus making it an effective tool for diagnostic purposes and useful for epidemiological and drug studies.

## Background

Malaria is one of the leading infectious diseases in the world, with 300–500 million clinical cases and 1–3 million deaths each year [1]. Traditionally diagnosis of malaria is based on microscopic detection of *Plasmodium *parasites in Giemsa-stained blood slides. In recent decades, antigen detection assays and molecular detection assays were introduced as alternatives to microscopy [2]. Antigen detection assays are mainly aimed at the identification of *Plasmodium falciparum*. Only very few assays are able to identify infections caused by other human *Plasmodium *species [2,3]. Furthermore, the sensitivity and specificity of these tests is low and parasite quantification is not possible [3,4]. The application of molecular techniques circumvents the limitations of conventional malaria diagnosis. PCR based assays are sensitive and can be converted to a quantitative format if SYBR green or molecular probes (e.g. a Taqman probe or a molecular beacon) are used in real time assays [5-7]. Alternatively, Real-time Quantitative Nucleic Acid Sequence Based Amplification (real-time QT-NASBA) technology can be applied, which has some advantages above real-time PCR assays. The real-time QT-NASBA assay is simple and fast compared to real-time PCR assays that can take up to four hours compared to 60 minutes in the case of NASBA [8,9]. Furthermore, real-time QT-NASBA detects ribosomal RNA of which more copies are present per genome in a parasite than the corresponding DNA on which PCR is based. This makes NASBA a very sensitive diagnostic assay. Moreover, NASBA is based on an isothermal reaction at 41 degrees that does not require a DNA denaturing step hereby preventing amplification of genomic DNA in case of contamination [8]. Real-time QT-NASBA, using a molecular beacon as detection probe, has been developed for *P. falciparum *and has shown to be very sensitive with a detection limit of 20 parasites/ml [9]. Detection of the other parasites causing human malaria, i.e. *Plasmodium vivax, Plasmodium malariae *and *Plasmodium ovale*, is of clinical importance in order to decide on appropriate treatment. This paper describes the development of a real-time QT-NASBA for the detection, identification and quantification of these *Plasmodium *species.

## Methods

### Primer/probe selection and in vitro RNA production

**Figure 1:**
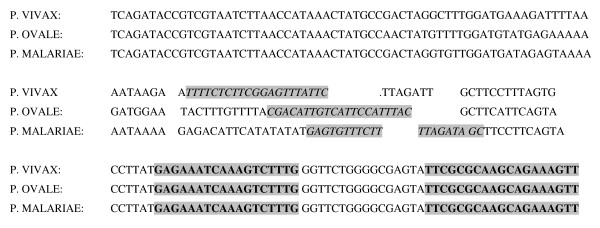
**Alignment of sequenced 18SrRNA gene**. Forward primers used in the different assays are shown in italics. Beacon and reverse primers are shown in bold.

Generic *Plasmodium *forward (5'-TCAGATACCGTCGTAATCTTA-3') and reverse (5'-TTCGCGCAAGCAGAAAGTT-3') primers were used to amplify a 180 bp region of *P. vivax*, *P. ovale *and *P. malariae *18S rRNA gene by PCR as previously described [10]. The amplified fragments were cloned into plasmid pCR2 in *Escherichia coli *INValphaF (Invitrogen, Carlsbad, California USA). Next, *in vitro *RNA was produced with the transcription kit SP6/T7 (Roche, Mannheim, Germany) and the cloned fragment was sequenced (Base Clear, Leiden, The Netherlands). Production of *P. falciparum *18S rRNA based *in vitro *RNA was performed previously [10]. Primers were selected on the basis of the DNA sequences of the three species (Figure 1). Homology of these sequences with published sequences and specificity of primers was analysed with the BLAST database for homology search [15]. The NASBA forward primers were chosen on their specificity for the targeted species. *P. falciparum*: 5'GTCATCTTTCGACGTGACTT-3'; *P. vivax*: 5'-TTTCTCTTCGGAGTTTATTC-3'; *P. ovale*: 5'-CGACATTGTCATTCCATTTAC-3'; *P. malariae*: 5'-GAGTGTTTCTTTTAGATAGC-3'. A T7 promotor sequence was added to the generic *Plasmodium *NASBA reverse primer; 5'-AATTCTAATACGACTCACTATAGGGAGAAGGAACTTTCTCGCTTGCGCGAA-3'. The published pf18S molecular beacon 5'-6-carboxyfluorescein-CGATCGGAGAAATCAAAGTCTTTGGGCGATCG-dimethylaminoazosulfonic acid-3' was used as a detection probe [9].

The molecular work was performed under permit 02–080 granted on 22 February 2002 to KIT Biomedical Research by the Netherlands Ministry for Spatial Planning, Housing and the Environment.

### Real-time – QT-NASBA

Real-time QT-NASBA for 18S rRNA of *P. falciparum, P. vivax*, *P. malariae *and *P. ovale *was performed on an IQ5 Real-Time analyser (Bio-RAD). The reactions were performed with the Nuclisens Basic kit for amplification (BioMerieux) according to the manufacturers instructions with a KCl concentration of 80 mM for *P. falciparum, P. vivax *and *P. malariae *and 70 mM for *P. ovale*. The reaction mixture (5 μl) containing the primers (100 pmol/μl) molecular beacon (20 μM) and template RNA (2.5 μl) was incubated at 65°C for two minutes followed by two minutes at 41°C. Thereafter, 2.5 μl enzyme mixture from the basic kit was added to each reaction. Amplification was monitored for 60 minutes after which the results were analysed. A sample containing only water and reaction mixture was used as blank and served as control for background fluorescence. The signal produced by the blank samples is automatically subtracted from the analytical samples (Bio-RAD IQ5 software v. 1.0). In order to quantify the number of parasites in a clinical sample, a 10 fold serial dilution of 10^9 ^to 10^2 ^molecules of *in vitro *RNA per amplification reaction of each respective *Plasmodium *species was run in triplicate in each test, wherein 10^4 ^molecules corresponds to one *Plasmodium *parasite [10].

### Clinical samples

Blood samples for validation (n = 79) were obtained from patients diagnosed with malaria by detection and differentiation of *Plasmodium *parasites in Giemsa-stained slides with standard microscopy according to WHO recommendations [11] or PCR methods as established in the contributing medical centres. The samples were collected from returned travellers with clinical symptoms visiting the out patient clinic of the University Medical Centre Leiden (The Netherlands) and the Academic Medical Centre Amsterdam (The Netherlands) and from ongoing field studies in Vietnam, Turkey and Kenya (Table 1). The samples were tested blinded from the diagnostic results. Specificity of the assays was tested against *in vitro *RNA of the four *Plasmodium *species and RNA isolated from *Plasmodium berghei *isolates (12 samples). Furthermore 20 samples from Dutch healthy blood donors were tested as negative controls. Blood samples (50 μl) were mixed with 950 μl guanidium isothiocyanate lysis buffer and RNA was isolated as described previously [12].

**Table 1:** Overview of sample origin

Species	microscopy	Origin of samples
*P. falciparum*	11	Kenya (KIT^a^)
*P. vivax*	37	8 LUMC^b^, 24 Turkey (Izmir^c^), 5 Vietnam (KIT)
*P. malariae*	7	2 Kenya (KIT) 4 Kenya (Nijmegen^d^)
*P. ovale*	4	3 LUMC 1 AMC^e^
Mixed infection	20	Kenya (Nijmegen^d^)
*P. berghei*	12	LUMC (mouse isolate)

a. Royal Tropical Institute, Amsterdam, The Netherlands.

b. Leiden University Medical Centre, Leiden, The Netherlands.

c. Ege University Medical School, Izmir, Turkey.

d. Radboud University, Nijmegen, The Netherlands.

e. Academic Medical Centre, Amsterdam, The Netherlands

### Statistical analysis of test performance

Microscopy performed at initial diagnosis was considered as the golden standard for this purpose and all NASBA results were compared to these results. The agreement between microscopy and the real-time QT-NASBA assay was determined by calculating Kappa values with a 95% confidence interval (Altman, 1991). Kappa values express the agreement beyond chance and a kappa value of 0.21–0.60 is a moderate, a kappa value of 0.61–0.80 a good and kappa > 0.80 an almost perfect agreement beyond chance.

## Results

### Analytical performance of the assay

**Figure 2:**
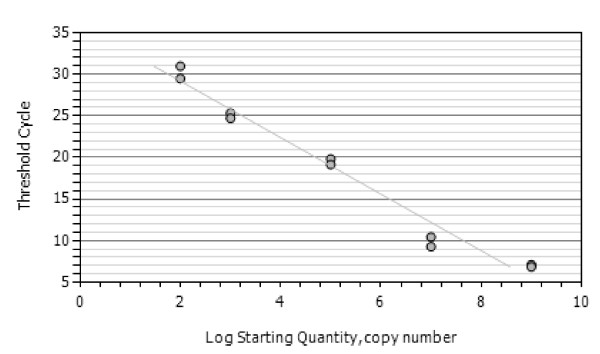
**Standard curves of the *P. malariae *assay**. A standard curve of the NASBA assay with *P. malariae *in vitro RNA using the *P. malariae *specific forward and generic revere primer and beacon. R^2 ^= 0,972.

**Figure 3:**
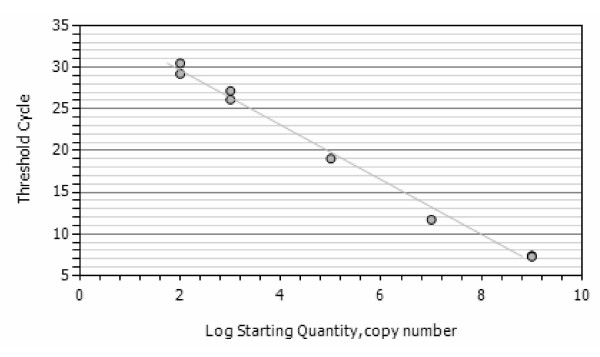
**Standard curves of the *P. vivax *assay**. A standard curve of the NASBA assay with *P. vivax *in vitro RNA using the *P. vivax *specific forward and generic revere primer and beacon wit an R^2 ^= 0,992.

**Figure 4:**
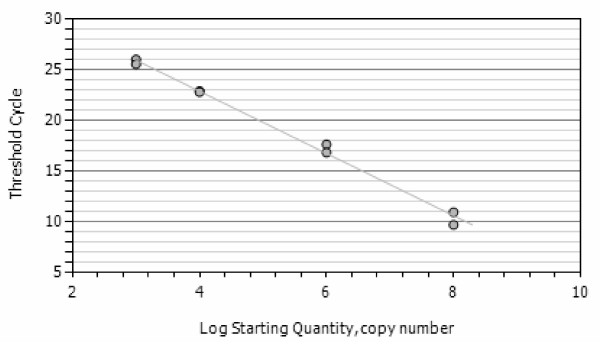
**Standard curves of the *P. ovale *assay **and figure c the standard curve of the NASBA assay with *P. ovale *in vitro RNA using the *P. ovale *specific forward and generic revere primer and beacon. R^2 ^= 0,993.

The lower detection limit of the developed assay as determined by analysing serial dilutions of *in vitro *RNA of *P. vivax*, *P. ovale *or *P. malariae*, respectively, was 100 – 1,000 molecules of *in vitro *RNA for each species, corresponding to 0.1 – 0.01 parasite per diagnostic sample (i.e. 50 μl of processed blood) (Figure 2, 3 and 4). No cross-hybridisation of the primers with the *in vitro *RNA of the other species and none with *P. berghei *RNA isolated from mice (n = 12) was observed. Blood samples form healthy donors (n = 20) showed no reaction in any of the assays.

### Clinical performance of the assay

The developed real-time QT-NASBA was further evaluated using 79 clinical samples (Table 2). Real-time QT-NASBA confirmed the initial diagnosis with microscopy of 69 out of the 79 clinical samples. The 37 *P. vivax *samples were found to be *P. vivax *with microscopy as well as with NASBA. However, one clinical sample gave an additional weak *P. ovale *signal with the real-time QT-NASBA. The four *P. ovale *samples were identified as such by real-time QT-NASBA. However, one sample gave a weak additional *P. vivax *signal. Two of the seven *P. malariae *mono-infections were found negative by NASBA. Furthermore, three of the seven *P. malariae *mono-infections were identified with real-time QT-NASBA as a *P. falciparum*/*P. malariae *mixed infection. Only 11 *P. falciparum *samples were analysed with real-time QT-NASBA in the present study, as a thorough analysis of this species has already been done [9]. The microscopical diagnosis *P. falciparum *of the clinical samples tested in the present study was confirmed by NASBA. Of the 20 *P. falciparum/P. malariae *mixed infections, 19 were identified as a mixed infection with NASBA: 17 being a mixed infection of *P. falciparum/P. malariae *and two samples gave a weak *P. vivax *reaction instead of *P. malariae*. One sample was identified with real-time QT-NASBA as a *P. falciparum *mono-infection.

**Table 2:** Positive *Plasmodium *samples.

Species	Microscopy	NASBA *Pf*	NASBA *Pv*	NASBA *Po*	NASBA *Pm*
*P. falciparum*	11	11	0	0	0
*P. vivax*	37	0	37	1	0
*P. malariae*	7	3	0	0	5
*P. ovale*	4	0	1	4	0
Mixed infection	20	20	2	0	17
*P. berghei*	12	0	0	0	0

The table gives an overview of the positive results of the 99 *Plasmodium *samples analyzed with microscopy and NASBA.

Of the 10 discordant results, seven samples were re-analysed by re-reading the blood slides by an expert microscopist who was blinded from the original microscopy and NASBA results. There were no back-up slides or PCR samples available from the other three discordant results. The microscopic re-analysis resolved five discordant results. In the three *P. malariae *mono-infections that were identified as a mixed infection by real-time QT-NASBA, a very low number of *P. falciparum *was found after re-reading the slides. In the two slides which were initially diagnosed by microscopy as being *P. falciparum/P. malariae *mixed infections, but with real-time QT-NASBA as being a *P. falciparum/P. vivax *mixed infection, the results of re-reading the blood slides were inconclusive for the presence of *P. malariae *and/or other *Plasmodium *species.

### Statistical analysis

A high degree of agreement was observed between the real-time QT-NASBA assay and microscopy in the present study. A kappa value of 0.926 (95% CI 0.872–0.967) indicates a very good agreement beyond change.

## Discussion

The aim of the present study was to develop a real-time QT-NASBA for the detection of all four human *Plasmodium *species. Based on the analytical evaluation of the developed test, it was concluded that the sensitivity of the developed tests is 0.1 to 0.01 parasites per diagnostic sample. This is comparable to the previously developed NASBA for *P. falciparum *[9] and approximately 50 times more sensitive than standard microscopy [2]. The developed molecular assays identified in 69 out of 79 samples the same species as the initial microscopical diagnosis. After re-checking the slides, the molecular diagnosis appeared to be correct in three discordant cases, leaving five results unresolved. The apparent discrepancy between the two diagnostic tests may be due to a very low number of parasites in the sample below the detection limit of microscopy, but still detectable with real-time QT-NASBA. The fact that in one sample no signal was obtained with real-time QT-NASBA could be due to degradation of the RNA or extraction failure. Unfortunately there was no backup sample available to repeat the extraction and analysis. In principal, quantification of parasites is possible with real-time QT-NASBA, but comparison with microscopic data from the clinical samples was not possible since parasite counts from the slides were not available. A parasite *in vitro *culture was also not available for *P. vivax, P. ovale *and *P. malariae*, making it difficult to make exact calculations. The parasite calculations used in the present study are based on the number of *in vitro *RNA molecules, which correlates to the number *P. falciparum *parasites [10]. It is assumed that these quantities are comparable to the other species. In general there is little clinical relevance for quantification of the *P. ovale*, *P. malariae *and *P. vivax *since the patients normally have < 2% parasitaemia [13] and treatment is given on basis of the infecting species and not on parasitaemia. In contrast, the parasitaemia in *P. falciparum *infection is an important factor for treatment regimen and monitoring of treatment efficacy. It has been shown that QT-NASBA is a valuable tool for assessing the parasite dynamics in studies where drug efficacy is monitored or drug combinations are compared [10,14]. The submicroscopic detection limit of the NASBA technique offers the possibility to monitor even small differences that are otherwise not noticed by microscopy and may even be a predictor for treatment failure [14].

## Conclusion

The developed real-time QT-NASBA for the detection of all four human *Plasmodium *species based on the 18S rRNA gene of *Plasmodium *showed to be a very sensitive and specific technique with a detection limit of 0.1 parasites per diagnostic sample. The assay can be used for the detection, identification and quantitative measurement of all human *Plasmodium *species even at low parasite levels, thus making it an effective tool for diagnostic purposes and useful for epidemiological and drug studies.

## Authors' contributions

PFM: Development and design of the NASBA assays, validation of the assay and molecular analysis of samples. Analysis and interpretation of the data, drafting and preparing the manuscript.

GJS: Cloning and expression of in vitro RNA, Sequencing of *Plasmodium *templates and primer design and molecular analysis.

PAK: Facilitating and coordination of the collection of clinical samples. Interpretation of the data and critically revising the manuscript.

HDFHS: Conception of the study and participation in its design and coordination. Interpetation of the data, critically reading of the manuscript

All authors read and approved the final manuscript.

## References

[B1] World Health Organisation (1991). WHO/CDS/RBM/2000.14. malaria Diagnosis New Perspectives. WHO/MAL/20001091-WHO Basic malaria microscopy – part 1.

[B2] Makler MT, Palmer CJ, Ager AL (1998). A review of practical techniques for the diagnosis of malaria. Ann Trop Med Parasitol.

[B3] Moody A (2002). Rapid diagnostic tests for malaria parasites. Clin Microbiol Rev.

[B4] Tham JM, Lee SH, Tan TM, Ting RC, Kara UA (1999). Detection and species determination of malaria parasites by PCR: comparison with microscopy and with ParaSight-F and ICT malaria Pf tests in a clinical environment. J Clin Microbiol.

[B5] Perandin F, Manca N, Calderaro A, Piccolo G, Galati L, Ricci L, Medici MC, Arcangeletti MC, Snounou G, Dettori G, Chezzi C (2004). Development of a real-time PCR assay for detection of *Plasmodium falciparum*, *Plasmodium vivax*, and *Plasmodium ovale *for routine clinical diagnosis. J Clin Microbiol.

[B6] Rougemont M, Van Saanen M, Sahli R, Hindrikson HP, Bille J, Jaton K (2004). Detection of Four *Plasmodium *species in blood from humans by 18S rRNA gene subunit-based and Species-Specific Real-Time PCR Assays. J Clin Microbiol.

[B7] Andrews L, Andersen RF, Webster D, Dunachie S, Walther RM, Bejon P, Hunt-Cooke A, Bergson G, Sanderson F, Hill AV, Gilbert SC (2005). Quantitative real-time polymerase chain reaction for malaria diagnosis and its use in malaria vaccine clinical trials. Am J Trop Med Hyg.

[B8] Cools I, Uyttendale M, D'Haese E, Nelis HJ, Debevere J Development of a real-time NASBA assay for the detection of *Campylobacter jejuni *cells. J Microbiol Methods.

[B9] Schneider P, Wolters L, Schoone G, Schallig H, Sillekens P, Hermsen R, Sauerwein R (2005). Real-time nucleic acid sequence-based amplification is more convenient than Real-Time PCR for quantification of *Plasmodium falciparum*. J Clin Microbiol.

[B10] Schoone GJ, Oskam L, Kroon NCM, Schallig HDFH, Omar SA (2000). Detection and quantification of *Plasmodium falciparum *in blood samples using quantitative nucleic acid sequence based amplification. J Clin Microbiol.

[B11] World Health Organization and UNICEF (2005). World Malaria report 2005. WHO/HTM/MAL/20051102.

[B12] Boom R, Sol CJ, Salimans MM, Jansen CL, Wertheim-van Dillen PM, Van den Noorda J (1990). Rapid and simple method for purification of nucleic acids. J Clin Microbiol.

[B13] Gilles HM, Warrell DA (1993). Bruce-Chwatt's Essential Malariology.

[B14] Omar SA, Mens PF, Schoone GJ, Yusuf A, Mwangi J, Kaniaru S, Omer GA, Schallig HDFH (2005). *Plasmodium falciparum: *evaluation of a quantitative nucleic acid sequence-based amplification assay to predict the outcome of sulfadoxine-pyrimethamine treatment of uncomplicated malaria. Exp Parasitol.

[B15] NCBI BLAST homology search. http://www.ncbi.nlm.nih.gov/blast/.

